# Supplementary material for: Missed Cystic Fibrosis Newborn Screening Cases due to Immunoreactive Trypsinogen Levels below Program Cutoffs: A National Survey of Risk Factors
Source: Int J Neonatal Screen. 2022 Oct 27;8(4):58. doi: 10.3390/ijns8040058 (PMC9680406; doi:10.3390/ijns8040058)
Supplement: Supplementary file 1 [file IJNS-08-00058-s001.zip › 1. Section S1 Information about your State Cystic Fibrosis Newborn Screening Program.pdf]

Section 1. Information about your State's Cystic Fibrosis Newborn Screening Program

1. Name and contact information of person coordinating the collection of data for this survey and names of other staff who should be acknowledged.

   a. Name

   b. Title

   c. Phone

   d. email

   e. Address

   City State Zip

   f. Names of other staff to be acknowledged

2. Name of your state
3. Date your state started its cystic fibrosis newborn screening program)
4. Type of CF NBS screening method your state used on 12/31/12 (e.g., IRT-IRT, IRT-DNA, IRT-IRT-DNA, IRT-DNA-Sequencing)
5. Date your state first started using the screening method in Question 4)
6. Complete name of the IRT assay your state used on 12/31/12 [e.g., DELFIA® Neonatal IRT Kit (S&S 903) Product # A005-110; AutoDELFIA® Neonatal IRT Kit (S&S 903) Product # B005-112]
7. Date your state first started using the IRT assay in Question 6
8. Latest date you answered for Questions 5 and 7 (e.g., 12/02/09 is later than 12/03/08)

IMPORTANT NOTE: Please answer all subsequent questions referring only to the time period starting with the date in Question 8 and ending on 12/31/12.

9. Total Number of CF Cases that were identified below the IRT cut off value _______
   (***NOTE:*** *Complete the questions in Section 2 for each CF case below the IRT cut off value.*)

10. Describe the newborn blood spot handling protocol used in your state's hospitals with particular reference to the type of filter paper used, age at blood spot collection, drying method and location, the packaging of specimens, and any precautionary steps taken to keep the specimens from heat and humidity

11. Describe the method(s) used to ship blood spots from the hospitals to your state's IRT testing laboratory(ies) with particular reference to the shipping container, shipper or currier, length of time en route, and any precautionary steps taken to keep the specimens from heat and humidity

12. Describe the blood spot handling protocol in your State lab(s) with a focus on maintenance of temperature and humidity controls (e.g., do you place the specimens in a refrigerator prior to IRT testing?)

13. Describe the details of the IRT cutoff(s) that is used in your NBS program. In particular, whether it is fixed or floating; based on a percentile or a value; and the specific methods used to calculate floating cutoffs? If your state is a 2-specimen state, please include information about both specimens.

14. Are the newborn blood spots collected during this period available for research purposes, and if so, until what calendar date (or age of child) and are they banked in a temperature-controlled environment?

15. After completion of all sections of the survey, please provide any clarifications below:
