# Supplementary material for: Missed Cystic Fibrosis Newborn Screening Cases due to Immunoreactive Trypsinogen Levels below Program Cutoffs: A National Survey of Risk Factors
Source: Int J Neonatal Screen. 2022 Oct 27;8(4):58. doi: 10.3390/ijns8040058 (PMC9680406; doi:10.3390/ijns8040058)
Supplement: Supplementary file 1 [file IJNS-08-00058-s001.zip › 2. Section S2 CF Cases Below the IRT Cut Off Value.pdf]

## **Section 2.** CF Cases Below the IRT Cut Off Value

**IMPORTANT NOTE:** Complete a separate Section 2 (Questions 16-46) for each CF case in Question 9 below the IRT cutoff value during the time period starting with the date in Question 8 and ending on 12/31/12.

Case *(State's coded reference number for tracking purposes)* ______________________________

**16.** Child' sex ☐ M ☐ F ☐ Unk

**17.** Was child part of a multiple birth? ☐ Y ☐ N ☐ Unk

**18.** Child's race: White? ☐ Y ☐ N ☐ Unk

**19.** Child's race: Black? ☐ Y ☐ N ☐ Unk

**20.** Child's race: other? ☐ Y ☐ N ☐ Unk

**21.** Child's ethnicity: Hispanic? ☐ Y ☐ N ☐ Unk

**22.** Hospital of birth Name ______________________________

City ______________________________ State ________

**23.** Day of week birth occurred ☐ Sun ☐ M ☐ T ☐ W ☐ Th ☐ F ☐ Sat ☐ Unk

**24.** Gestational age at birth _______ weeks _______ days

**25.** Birth weight _______ grams

**26.** Meconium ileus ☐ Y ☐ N ☐ Unk

**27.** CFTR mutations genotype *(mutation 1/mutation 2; TG Tract Poly T 1/TG Tract Poly T 2)*

______________________________

**28.** Age at CF diagnosis _______ days

**29.** Sweat chloride test value *(list highest)* ______ mEq/L

**30.** CF diagnosis suggested by family history? ☐ Y ☐ N ☐ Unk

**31.** CF diagnosis suggested by symptoms? ☐ Y ☐ N ☐ Unk

**32.** CF diagnosis suggested by CF prenatal/carrier screening? ☐ Y ☐ N ☐ Unk

**33.** List any non-CF disorders that were present ______________________________

| | Specimen 1 | Specimen 2 | Retest |
|---|---|---|---|
| **34.** Age of child at specimen collection | _______ hours | _______ hours | _______ hours |
| **35.** Age of child at time of IRT analysis | _______ days | _______ days | _______ days |
| **36.** Month of specimen collection | ____________ | ____________ | ____________ |
| **37.** IRT value | _______ ng/mL | _______ ng/mL | _______ ng/mL |
| **38.** IRT value cut point used | _______ ng/mL | _______ ng/mL | _______ ng/mL |
| **39.** IRT percentile | _______ | _______ | _______ |
| **40.** IRT percentile cut point used | _______ | _______ | _______ |
| **41.** IRT Kit Lot Number | ____________ | ____________ | ____________ |
| **42.** IRT Kit Lot median value | _______ ng/mL | _______ ng/mL | _______ ng/mL |

### Questions 43-46 refer to the period from birth to the time of specimen collection

| | | | |
|---|---|---|---|
| **43.** Infant feedings *(select one)* | ☐ breast only ☐ Unk<br>☐ formula only<br>☐ breast and formula | ☐ breast only ☐ Unk<br>☐ formula only<br>☐ breast and formula | ☐ breast only ☐ Unk<br>☐ formula only<br>☐ breast and formula |
| **44.** Red blood cell transfusion | ☐ Y ☐ N ☐ Unk | ☐ Y ☐ N ☐ Unk | ☐ Y ☐ N ☐ Unk |
| **45.** Intravenous nutrition | ☐ Y ☐ N ☐ Unk | ☐ Y ☐ N ☐ Unk | ☐ Y ☐ N ☐ Unk |
| **46.** Neonatal Intensive Care Unit | ☐ Y ☐ N ☐ Unk | ☐ Y ☐ N ☐ Unk | ☐ Y ☐ N ☐ Unk |
