# Supplementary material for: Missed Cystic Fibrosis Newborn Screening Cases due to Immunoreactive Trypsinogen Levels below Program Cutoffs: A National Survey of Risk Factors
Source: Int J Neonatal Screen. 2022 Oct 27;8(4):58. doi: 10.3390/ijns8040058 (PMC9680406; doi:10.3390/ijns8040058)
Supplement: Supplementary file 1 [file IJNS-08-00058-s001.zip › 3. Section S3 CF Cases At or Above the IRT Cut Off Value.pdf]

Section 3. CF Cases At or Above the IRT Cut Off Value

IMPORTANT NOTE: Please answer all questions referring only to the time period starting with the date in Question 8 and ending on 12/31/12.

For questions 47-74, provide the number of CF cases at or above the IRT cut-off value in each category. If your state does not collect the requested data item, enter "DC" in the "Unkown" field.

47. Total CF Cases at or above IRT cut off *(see instructions for Section 3 for definition)* _______

48. Child's sex Male _______ Female _______ Unknown _______

49. Child's race: White Yes _______ No _______ Unknown _______

50. Child's race: Black Yes _______ No _______ Unknown _______

51. Child's race: Other Yes _______ No _______ Unknown _______

52. Child's ethnicity Hispanic _______ Non-Hispanic _______ Unknown _______

53. Hospital of Birth *(Complete all but last column in Worksheet 1 with the number of CF Cases at or above the IRT cut-off in each hospital.)*

54. Day of week birth occurred

Sun _______ Mon _______ Tue _______ Wed _______

Thu _______ Fri _______ Sat _______ Unknown _______

55. Gestational age at birth (completed weeks)

<34 _______ 35-36 _______ 37-41 _______ >41 _______ Unknown _______

56. Birth weight (grams) <1500 _______ 1500-2499 _______ 2500+ _______ Unknown _______

57. Meconium ileus Yes _______ No _______ Unknown _______

58. CFTR mutations

Not genotyped _______

Genotyped *(provide count of each genotype on Worksheet 2)* _______

Unknown if genotyped _______

59. Age at CF diagnosis (days)

<29 _______ 29-56 _______ 57-84 _______ 85-112 _______ 113-130 _______

131-158 _______ >158 _______ Unknown _______

60. CF diagnosis suggested by newborn screening Yes _______ No _______ Unknown _______

61. CF diagnosis suggested by family history Yes _______ No _______ Unknown _______

62. CF diagnosis suggested by symptoms Yes _______ No _______ Unknown _______

63. CF diagnosis suggested by CF prenatal/carrier screening Yes _______ No _______ Unknown _______

For states using a 2-specimen IRT-IRT or IRT-IRT-DNA screening model, Questions 62-75 should be answered for both specimens, as specified in the table. Provide information only for the specimen(s) used to make the CF NBS interpretation.

	Specimen 1	Specimen 2
**64.** Median age of children at specimen collection	_______ hours	_______ hours
**65.** Median age of children at time of IRT analysis	_______ days	_______ days

66. Month of specimen collection

Specimen 1: Jan _______ Feb _______ Mar _______ Apr _______ May _______ June _______

July _______ Aug _______ Sep _______ Oct _______ Nov _______ Dec _______

Specimen 2: Jan _______ Feb _______ Mar _______ Apr _______ May _______ June _______

July _______ Aug _______ Sep _______ Oct _______ Nov _______ Dec _______

	Specimen 1	Specimen 2
**67.** Median IRT value	_______ ng/mL	_______ ng/mL
**68.** IRT value cut point used *(provide median, if multiple cut points used)*	_______ ng/mL	_______ ng/mL
**69.** Median IRT percentile	_______	_______
**70.** IRT percentile cut point used *(provide median, if multiple cut points used)*	_______	_______

Questions 71-74 refer to the period from birth to the time of specimen collection:

	Specimen 1	Specimen 2
**71.** Infant Feedings	_______ Breast only	_______ Breast only
	_______ Formula only	_______ Formula only
	_______ Breast and formula	_______ Breast and formula
	_______ Unknown	_______ Unknown
**72.** Red Blood Cell Transfusion	_______ Yes	_______ Yes
	_______ No	_______ No
	_______ Unknown	_______ Unknown
**73.** Intravenous Nutrition	_______ Yes	_______Yes
	_______ No	_______ No
	_______ Unknown	_______ Unknown
**74.** Neonatal Intensive Care Unit	_______ Yes	_______ Yes
	_______ No	_______ No
	_______ Unknown	_______ Unknown
