# Supplementary material for: Missed Cystic Fibrosis Newborn Screening Cases due to Immunoreactive Trypsinogen Levels below Program Cutoffs: A National Survey of Risk Factors
Source: Int J Neonatal Screen. 2022 Oct 27;8(4):58. doi: 10.3390/ijns8040058 (PMC9680406; doi:10.3390/ijns8040058)
Supplement: Supplementary file 1 [file IJNS-08-00058-s001.zip › 4. Section S4 All Screened Newborns.pdf]

Section 4. All Screened Newborns

IMPORTANT NOTE: Please answer all questions referring only to the time period starting with the date in Question 8 and ending on 12/31/12.

For questions 75-96, provide the number of newborns receiving IRT testing in each category. If your state does not collect the requested data item, enter "DC" in the "Unknown" field.

75. Total newborns receiving IRT testing ______________________

76. Child's sex Male ____________ Female ____________ Unknown ____________

77. Child's race: White Yes ____________ No ____________ Unknown ____________

78. Child's race: Black Yes ____________ No ____________ Unknown ____________

79. Child's race: Other Yes ____________ No ____________ Unknown ____________

80. Child's ethnicity Hispanic ___________ Non-Hispanic ___________ Unknown ___________

81. Hospital of birth *(Complete Worksheet 1, last column with the number of newborns receiving IRT Testing in each hospital.)*

82. Day of week birth occurred

Sun _________ Mon _________ Tue _________ Wed _________

Thu _________ Fri _________ Sat _________ Unknown _________

83. Gestational age at birth (completed weeks)

<34 _________ 35-36 _________ 37-41 _________ >41 _________ Unknown _________

84. Birth weight (grams)

<1500 ___________ 1500-2499 ___________ 2500+ ___________ Unknown ___________

For states using a 2 specimen IRT-IRT or IRT-IRT-DNA screening model, Questions 85-96 should be answered for both specimens, as specified in the table.

	Specimen 1	Specimen 2
**85.** Median age of children at specimen collection	______ hours	______ hours
**86.** Median age of children at time of IRT analysis	______ days	______ days

87. Month of specimen collection

Specimen 1: Jan _______ Feb _______ Mar _______ Apr _______ May _______ June _______

July _______ Aug _______ Sep _______ Oct _______ Nov _______ Dec _______

Specimen 2: Jan _______ Feb _______ Mar _______ Apr _______ May _______ June _______

July _______ Aug _______ Sep _______ Oct _______ Nov _______ Dec _______

	Specimen 1	Specimen 2
**88.** Median IRT value	_______ ng/mL	_______ ng/mL
**89.** Median IRT value cut point used	_______ ng/mL	_______ ng/mL
**90.** Median IRT percentile	_______	_______
**91.** Median IRT percentile cut point used	_______	_______

Questions 92-95 refer to the period from birth to the time of specimen collection:

	Specimen 1	Specimen 2
**92.** Infant Feedings	____________ Breast only	____________ Breast only
	____________ Formula only	____________ Formula only
	____________ Breast and formula	____________ Breast and formula
	____________ Unknown	____________ Unknown
**93.** Red Blood Cell Transfusion	____________ Yes	____________ Yes
	____________ No	____________ No
	____________ Unknown	____________ Unknown
**94.** Intravenous Nutrition	____________ Yes	____________ Yes
	____________ No	____________ No
	____________ Unknown	____________ Unknown
**95.** Neonatal Intensive Care Unit	____________ Yes	____________ Yes
	____________ No	____________ No
	____________ Unknown	____________ Unknown

96. Information on IRT Kit Lots used *(Complete Worksheet 3)*
