# Supplementary material for: Missed Cystic Fibrosis Newborn Screening Cases due to Immunoreactive Trypsinogen Levels below Program Cutoffs: A National Survey of Risk Factors
Source: Int J Neonatal Screen. 2022 Oct 27;8(4):58. doi: 10.3390/ijns8040058 (PMC9680406; doi:10.3390/ijns8040058)
Supplement: Supplementary file 1 [file IJNS-08-00058-s001.zip › 5. Worksheet S1 Hospital Information.pdf]

Worksheet 1. Hospital Information

IMPORTANT NOTE: See instructions for Worksheet 1; use additional sheets as needed.

Hospital Code	Name/Address		Number of CF cases at or above cut-off from Section 3	Number of newborns recieving IRT testing from Section 4
____	Name ____		____	____
	City ____	State ____		
____	Name ____		____	____
	City ____	State ____		
____	Name ____		____	____
	City ____	State ____		
____	Name ____		____	____
	City ____	State ____		
____	Name ____		____	____
	City ____	State ____		
____	Name ____		____	____
	City ____	State ____		
____	Name ____		____	____
	City ____	State ____		
____	Name ____		____	____
	City ____	State ____		
____	Name ____		____	____
	City ____	State ____		
____	Name ____		____	____
	City ____	State ____		
____	Name ____		____	____
	City ____	State ____		
