# Supplementary material for: Missed Cystic Fibrosis Newborn Screening Cases due to Immunoreactive Trypsinogen Levels below Program Cutoffs: A National Survey of Risk Factors
Source: Int J Neonatal Screen. 2022 Oct 27;8(4):58. doi: 10.3390/ijns8040058 (PMC9680406; doi:10.3390/ijns8040058)
Supplement: Supplementary file 1 [file IJNS-08-00058-s001.zip › 6. Worksheet S2 CFTR Mutations.pdf]

Worksheet 2. CFTR Mutations

See instructions for Worksheet 2.

CFTR Genotype *(mutation 1/mutation 2; TG Tract-Poly T 1/TG Tract-Poly T 2)*	Number
F508del/F508del	________
F508del/G542X	________
F508del/R117H; (TG)11-9T/(TG)13-5T	________
F508del/R117H; (TG)11-9T/(TG)12-5T	________
F508del/R117H; Unknown TG Tract-Poly T	________
F508del/Unknown	________
Unknown/Unknown	________

(please list all other genotypes below; use additional sheets as needed)

____	________
____	________
____	________
____	________
____	________
____	________
____	________
____	________
____	________
____	________
____	________
____	________
____	________
____	________
____	________
____	________
____	________
____	________
____	________
____	________

Worksheet 2. CFTR Mutations

See instructions for Worksheet 2.

CFTR Genotype *(mutation 1/mutation 2; TG Tract-Poly T 1/TG Tract-Poly T 2)*	Number
