# Supplementary material for: Missed Cystic Fibrosis Newborn Screening Cases due to Immunoreactive Trypsinogen Levels below Program Cutoffs: A National Survey of Risk Factors
Source: Int J Neonatal Screen. 2022 Oct 27;8(4):58. doi: 10.3390/ijns8040058 (PMC9680406; doi:10.3390/ijns8040058)
Supplement: Supplementary file 1 [file IJNS-08-00058-s001.zip › 7. Worksheet S3 Information about IRT Kit Lots.pdf]

Worksheet 3. Information about IRT Kit Lots

See instructions for Worksheet 3; use additional sheets as needed.

IRT Kit Lot #	Dates used	Specimen (check one)	Number of newborns tested in lot from Section 4	IRT lot Median value	Number of CF Cases from Section 3
		☐ Specimen 1 ☐ Specimen 2		ng/mL	
		☐ Specimen 1 ☐ Specimen 2		ng/mL	
		☐ Specimen 1 ☐ Specimen 2		ng/mL	
		☐ Specimen 1 ☐ Specimen 2		ng/mL	
		☐ Specimen 1 ☐ Specimen 2		ng/mL	
		☐ Specimen 1 ☐ Specimen 2		ng/mL	
		☐ Specimen 1 ☐ Specimen 2		ng/mL	
		☐ Specimen 1 ☐ Specimen 2		ng/mL	
		☐ Specimen 1 ☐ Specimen 2		ng/mL	
		☐ Specimen 1 ☐ Specimen 2		ng/mL	
		☐ Specimen 1 ☐ Specimen 2		ng/mL	
		☐ Specimen 1 ☐ Specimen 2		ng/mL	
		☐ Specimen 1 ☐ Specimen 2		ng/mL	
		☐ Specimen 1 ☐ Specimen 2		ng/mL	
		☐ Specimen 1 ☐ Specimen 2		ng/mL	
		☐ Specimen 1 ☐ Specimen 2		ng/mL	
		☐ Specimen 1 ☐ Specimen 2		ng/mL	
		☐ Specimen 1 ☐ Specimen 2		ng/mL	
		☐ Specimen 1 ☐ Specimen 2		ng/mL	
		☐ Specimen 1 ☐ Specimen 2		ng/mL	
		☐ Specimen 1 ☐ Specimen 2		ng/mL	
