# Supplementary material for: Missed Cystic Fibrosis Newborn Screening Cases due to Immunoreactive Trypsinogen Levels below Program Cutoffs: A National Survey of Risk Factors
Source: Int J Neonatal Screen. 2022 Oct 27;8(4):58. doi: 10.3390/ijns8040058 (PMC9680406; doi:10.3390/ijns8040058)
Supplement: Supplementary file 1 [file IJNS-08-00058-s001.zip › 8. Instructions for all Sections and Worksheets.pdf]

State Survey

IRT levels and other characteristics of persons with false negative newborn screening tests for cystic fibrosis in the United States

(12/16/14)

INSTRUCTIONS FOR ALL SECTIONS

General - All Sections

This survey should be completed by one or more staff who have intimate knowledge of the cystic fibrosis newborn screening program in your state. The survey has four sections and three worksheets with specific instructions. Complete all sections by printing hardcopies and handwriting answers or by entering data directly into the PDF documents. Project staff may contact you as necessary during completion of this project.

This survey is requesting information about your cystic fibrosis newborn screening program. It is limited to a specific time period ending on 12/31/12. The start date will depend on when you began using the CF NBS algorithm (e.g., IRT/DNA) and IRT assay that were in use on 12/31/12. The start date for most programs will be the date your lab began conducting IRT analysis in the production environment and will be based on that accession date.

Section 1. Information about your State's Cystic Fibrosis Newborn Screening Program

Please start by answering the questions in Section 1, which are about your state's newborn screening program, both generally and specifically for cystic fibrosis. If you have existing documents that can be appended with the requested information, please write "see Appendix 1, 2, 3, etc." and any additional notes for the answer in the space provided. Save PDF file using the following nomenclature (STATE_XX_SECTION_1_DATE_YYMMDD.PDF) where 'XX' is your state code and 'YYMMDD' is the date saved in Year, Month, Day format.

Section 2. CF Cases Below the IRT Cut Off Value

The questions in Section 2 are about individual cases of CF who had IRT levels that were below your state's cut off value(s) during the time period starting with the date in Question 8 and ending on 12/31/12. Please include all low IRT cases regardless of how the CF diagnosis was made. Please confirm with your CF Follow Up Care Centers

that your list is complete for this time period. For states using a 2-specimen IRT/IRT screening model, Questions 34-46 should be answered for both specimens, as specified in the table. Provide information in Specimen 1 and 2 fields only for the specimen(s) used to make the CF NBS interpretation. If any additional specimens were tested, please provide that information under the Retest column. Complete Section 2 for each low IRT case during the time period by either printing blank copies and completing them by hand or saving separate Section 2 files for each case using the following nomenclature (STATE_XX_SECTION_2_CASE_YY.PDF) where 'XX' is your state code and 'YY' is the case number. Please label each case with a number, e.g., Case 01, Case 02, etc. and use a reference code for your own tracking purposes (Do not use names). If your state does not collect the requested data item, please leave it blank.

Section 3. CF Cases At or Above the IRT Cut Off Value

The questions in Section 3 require summary data on the number of cases of CF who had IRT levels that were at or above your state's cut off value(s) during the time period starting with the date in Question 8 and ending on 12/31/12. Please include all screen positive cases and those who were false negative cases because their CF mutations were missed by your state's testing panel. If your state does not collect the requested data item, please indicate Don't Collect ("DC") in the Unknown field. The sum of the numbers in each question should equal the total in Question 47. Please make a note in Question 15 in Section 1 if this is not true. Save PDF file using the following nomenclature (STATE_XX_SECTION_3_DATE_YYMMDD.PDF) where 'XX' is your state code and 'YYMMDD' is the date saved in Year, Month, Day format.

Section 4. All Screened Newborns

The questions in Section 4 require summary data on the number of newborns that got IRT testing during the time period starting with the date in Question 8 and ending on 12/31/12. If your state does not collect the requested data item, please indicate Don't Collect ("DC")in the Unknown field. The sum of the numbers in each question should equal the total in Question 75. Please make a note in Question 15 in Section 1 if this is not true. Save PDF file using the following nomenclature (STATE_XX_SECTION_4_DATE_YYMMDD.PDF) where 'XX' is your state code and 'YYMMDD' is the date saved in Year, Month, Day format.

Worksheet 1. Hospital Information

The purpose of this worksheet is to collect summary data on the number of CF cases from Section 3 and the number of newborns in Section 4 by hospital of birth. Save PDF file using the following nomenclature (STATE_XX_WORKSHEET_1_PAGE_YY_DATE_YYMMDD.PDF) where 'XX' is your state code, 'YY' is the page number and 'YYMMDD' is the date saved in Year, Month, Day format. It is acceptable to provide this information in a different form (such as SAS output file, spreadsheet or printout), if desired.

Worksheet 2. CFTR Mutations

The purpose of this worksheet is to collect summary data on the number of CF cases from Section 3 by CFTR genotype. Include counts of cases with 1 or 2 unknown mutations regardless of the type of genotype testing conducted. Provide IVS 8 Poly T, TG Tract, if known. Save PDF file using the following nomenclature (STATE_XX_WORKSHEET_2_PAGE_YY_DATE_YYMMDD.PDF) where 'XX' is your state code, 'YY' is the page number and 'YYMMDD' is the date saved in Year, Month, Day format. Use the additional PDF sheet after the first page is completed, as needed. It is acceptable to provide this information in a different form (such as SAS output file, spreadsheet or printout), if desired.

Worksheet 3. Information about IRT Kit Lots

The purpose of this worksheet is to collect summary data on the number of newborns in Section 4 and the number of CF cases from Section 3 by IRT Kit Lot. For each kit lot, provide dates used and median IRT value. For states using a 2-specimen IRT/IRT or IRT'IRT'DNA screening model, provide this information separately for Specimen 1 and Specimen 2. Save PDF file using the following nomenclature (STATE_XX_WORKSHEET_3_PAGE_YY_DATE_YYMMDD.PDF) where 'XX' is your state code, 'YY' is the page number and 'YYMMDD' is the date saved in Year, Month, Day format. It is acceptable to provide this information in a different form (such as SAS output file, spreadsheet or printout), if desired.

Questions and completed surveys can be addressed to:

Martin Kharrazi, Ph.D.
California Department of Public Health
850 Marina Bay Parkway, Bldg. P, 3rd Floor
Richmond, CA 94804
Office: 510-412-1480
Fax: 510-412-1511
Marty.Kharrazi@cdph.ca.gov
